# Hierarchical 2D Cu-MOF@Graphene-Based Hybrids for Supercapacitor Electrodes

**DOI:** 10.3390/nano15211628

**Published:** 2025-10-25

**Authors:** Mengkun Yang, Yongqiang Zhang, Wenjie Li, Pingwei Ye, Yijing Nie, Maiyong Zhu, Sumin Li

**Affiliations:** 1 School of Materials Science & Engineering, Jiangsu University, Zhenjiang 212013, China; 2222305060@stmail.ujs.edu.cn (M.Y.); 2212005078@stmail.ujs.edu.cn (Y.Z.); 2222305089@stmail.ujs.edu.cn (W.L.); nieyijing@ujs.edu.cn (Y.N.); maiyongzhu@ujs.edu.cn (M.Z.); 2 State Key Laboratory of NBC Protection for Civilian, Beijing 102205, China

**Keywords:** Cu-MOF, GO-COOH, soft-template, supercapacitor

## Abstract

Recently, two-dimensional metal–organic framework (2D MOF) hybrids are attracting much attention in supercapacitors. However, their performance is limited by the insufficient utilization of active sites and poor conductivity. Notably, the full utilization of active sites highly depends on the fast transport and diffusion of ions. Here, a Cu-MOF@GO-COOH hybrid was constructed, with GO-COOH as the substrate, to promote electron transfer, and Meso-Cu-MOF@GO-COOH was further obtained by introducing mesopores inside nanosheets to optimize the transportation paths for ions. The GO-COOH substrate improves the capacitance by enhancing the surface capacitive behavior, while the mesopores improve the charge-storage capacity by enhancing the diffusive behavior. The as-obtained Meso-Cu-MOF@GO-COOH exhibits a higher capacitance of 292.5 F g^−1^ compared with Cu-MOF@GO-COOH (193.7 F g^−1^) and 2D Cu-MOF (141.4 F g^−1^) at a current density of 1 A g^−1^. Moreover, the prepared Cu-MOF@GO-COOH//AC device delivers a capacitance of 63 F g^−1^ (0.5 A g^−1^), an energy density of 27.7 Wh kg^−1^, and a power density of 496.8 W kg^−1^, showing a great potential for practical applications.

## 1. Introduction

Benefiting from their high-power density, rapid charge–discharge process, greenness, and long lifetime, supercapacitors are gaining great attention as next-generation energy storage devices. To enhance their electrochemical performance, various materials, including traditional materials (carbon-based, conducting polymers, and metal oxides) and novel nanomaterials (quantum dots, transition metal dichalcogenides, Mxenes, etc.), have been developed as supercapacitor electrodes [1,2]. Metal–organic frameworks (MOFs) are a novel porous material, formed via the coordination reaction between metal ions and organic ligands. Benefiting from their diverse pore architecture, high surface area, and tunable functionality, MOFs are being intensively investigated in energy storage [3,4,5,6,7,8], detection [9,10,11,12,13], sensing [14,15,16,17,18], and adsorption [19], etc. Notably, owing to the highly accessible active sites, two-dimensional MOFs (2D MOFs), which are prone to producing capacitance, are drawing much attention in the field of supercapacitor electrodes [20,21,22,23,24,25].

However, the electrochemical performance of 2D MOFs is restricted by their unsatisfactory conductivity, stacking problems, and insufficient utilization of active sites, limiting their application as energy-storing electrodes. To overcome these shortcomings, some conductive materials, such as graphene-based and Ti_3_C_2_T_x_ substrates, are preferred to construct MOF-based hybrids [26,27,28]. By combining the merits of 2D MOFs with those of conductive materials, the electrochemical performances of MOF hybrids have been certainly enhanced [29,30,31].

To further shorten the gap between the practical and theoretical performance of 2D MOFs, maximizing the utilization of active sites is imperative. Notably, the hierarchically porous architecture has been proved to be an effective strategy to enhance electrode performance by promoting ion transport and diffusion [32,33,34,35,36]. By promoting the accessibility of ions to micropores and small-sized mesopores, the utilization of active sites is improved.

In this paper, to optimize the transport paths and to promote the movement of ions directly through 2D MOF nanosheets rather than around them, a soft template was used to produce mesopores inside MOF nanosheets, thus providing convenient channels for ion transport. Moreover, as a low-cost and environmentally friendly material, Cu-based MOFs present great potential in developing high-performance electrodes [37].

As an amphiphilic block copolymer, polystyrene-b-poly(ethylene oxide) presents a prominent merit, in that it has adjusting pore sizes, in which changing lengths of hydrophobic chain segments lead to various pores [38,39]. Here, a polystyrene-b-poly(ethylene oxide) (PS_102_-b-PEO_114_) was chosen as the soft template to produce mesopores inside MOF nanosheets. Benefiting from the ample carboxyl groups on the surface of GO-COOH, hydrogen bonds can be formed between GO-COOH and the micelles to promote micelles to arrange on the GO-COOH surface and to further guide the growth of Cu-MOF. After the micelles were removed, mesopores were left inside MOF nanosheets to obtain hierarchical Meso-Cu-MOFs@GO-COOH, in which new transport channels are provided for ions and their diffusion ability is enhanced, improving the effective utilization of active sites inside MOFs.

## 2. Experimental Methods

### 2.1. Synthesis of Materials

The synthetic methods of 2D Cu-MOF, GO-COOH, and BCP@GO-COOH are described in the Supplementary Materials Section.

Synthesis of Cu-MOF@GO-COOH. Firstly, 20 mg of Cu(OAc)_2_•H_2_O (>98%) was dissolved in a mixed solvent containing 2 mL of N, N-dimethylformamide (DMF, 99.5%), and 4 mL of ethanol absolute (EtOH, 99.7%). Next, 20 mg of H_2_BDC-NH_2_ (98.6%) and 10 mg of GO-COOH were added to another solvent containing 10 mL of DMF and 5 mL of EtOH to form a mixture. The copper–salt solution was slowly added to the mixture and stirred continuously for 30 min. After that, the product was collected, washed with DMF and EtOH, and dried at 60 °C for 24 h, to obtain Cu-MOF@GO-COOH.

**Figure 1:**
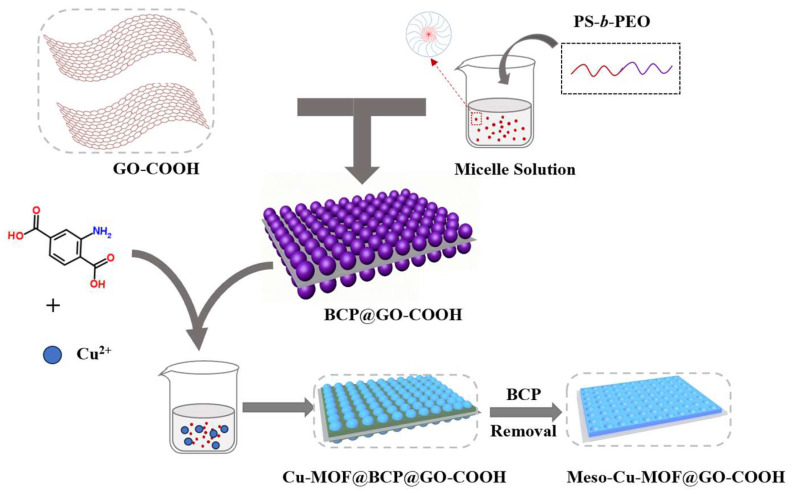
Schematic illustration of preparing Meso-Cu-MOF@GO-COOH.

Synthesis of Meso-Cu-MOF@GO-COOH. Firstly, 20 mg of Cu(OAc)_2_•H_2_O was dissolved in a mixture containing 2 mL of DMF and 4 mL of EtOH. Next, 20 mg of H_2_BDC-NH_2_ was added to the BCP@GO-COOH solution and stirred continuously for 60 min to form a mixture. Subsequently, the copper–salt solution was slowly added to the mixture and stirred continuously for 30 min. After that, the product was collected, and then washed with DMF, tetrahydrofuran (THF, 99.5%), and EtOH. At last, the as-obtained product was dried at 60 °C for 24 h to obtain Meso-Cu-MOF@GO-COOH (Figure 1).

Notably, the Cu-MOF@BCP@GO-GOOH can be obtained by washing with just DMF and EtOH, without THF.

### 2.2. Structural Analysis

Scanning electron microscopy (SEM, JEOL JXA-840A) and transmission electron microscopy (TEM, JEOL JEM-2100 PLUS) were used to analyze the surface morphology of samples. Fourier transforms infrared (FTIR) and X-ray diffraction (XRD) were performed to investigate the elemental composition of materials. The specific surface area and pore characteristic of samples were analyzed via a physisorption analyzer (ASAP 2020M).

### 2.3. Electrochemical Characterization

The electrochemical performances of samples were tested on an electrochemical workstation (CHI760E), including cyclic voltammetry (CV), galvanostatic charge–discharge (GCD), and electrochemical impedance spectroscopy (EIS). Using a three-electrode system to estimate the performance of single electrode, in which a working electrode (the as-prepared materials), counter electrode (Pt plate), and reference electrode (Ag/AgCl) were used. Additionally, 3 M KOH aqueous solution was used as the electrolyte.

The specific capacitance of electrode was calculated based on the following formula [40]:

*C* (F g^−1^) = *I* ∫ (1/*m* × *V*(*t*)) dt = *I* Δ*t*/*m* Δ*V* (1)

in which *I*, Δ*t*, and Δ*V* correspond to the current, discharge time, and voltage, respectively, while *m* is the mass of active materials.

An asymmetric supercapacitor (ASC) was prepared using Meso-Cu-MOF@GO-COOH and activated carbon (AC) as electrodes. The preparation process of working electrodes is displayed in the Supplementary Materials. The loading mass of the active material was 1–1.5 mg cm^−2^. To maximize the performance of device, the optimal mass ratio of two electrodes was calculated according to the following formula:

(2) $$\frac{m_{+}}{m_{-}}=\frac{C_{-}\times \Delta V_{-}}{C_{+}\times \Delta V_{+}}$$

in which *m*, *C*, and Δ*V* correspond to the mass, specific capacitance, and voltage of the positive and negative electrodes, respectively. Moreover, the capacitance value of the as-assembled ASC device can also by estimated according to Equation (1), except, in that case, *m* would refer to the mass of two electrodes. The energy density (*E*) and power density (*P*) of the ASC device are calculated via the following formulas [41]:

(3) $$E=\frac{C\times {∆V}^{2}}{2\times 3.6}$$

(4) $$P=\frac{3600\times E}{∆t}$$

in which *E* is the energy density, *P* is the power density, Δ*t* is the discharge time, and Δ*V* is the potential window.

## 3. Results and Discussion

### 3.1. Structural Characteristics

**Figure 2:**
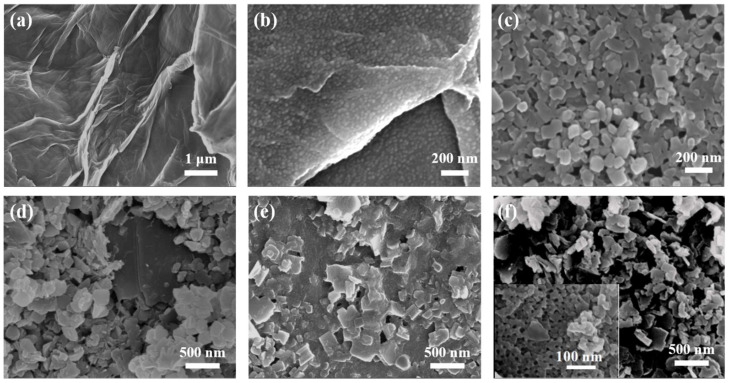
SEM images of samples: (**a**) GO-COOH, (**b**) BCP@GO-COOH, (**c**) 2D Cu-MOF, (**d**) Cu-MOF@GO-COOH, (**e**) Cu-MOF@BCP@GO-COOH, and (**f**) Meso-Cu-MOF@GO-COOH (the insert is high-resolution SEM image).

The microstructure of GO-COOH is shown in Figure 2a; many wrinkles can be noticed. Furthermore, when using GO-COOH as the substrate, promoted by the hydrogen bonding force, the soft template (PS_102_-b-PEO_114_) self-assembled into uniform spherical micelles (Figure 2b), and covered the surface of GO-COOH substrate. Notably, when the length of the hydrophobic chain segment in PS_n_-b-PEO_114_ (*n* = 70, 90, 102 and 150) was changed, micelles with different sizes were obtained (Figure S1), by which the sizes of mesopores can be regulated. Figure 2c displays the morphology of pristine 2D Cu-MOF, in which uniform nanosheets with a size of 100~200 nm can be seen. For the Cu-MOF@GO-COOH sample, the size of the nanosheets (Figure 2d) is somewhat larger than that of the pristine 2D Cu-MOF, presenting an average size of about 300 nm, which may be related to the induced growth originating from the functional groups on the GO-COOH surface (Figure S2). When using BCP@GO-COOH as the substrate to prepare 2D Cu-MOF, large numbers of spherical micelles could still be identified (Figure 2e). During the preparation of MOF nanosheets, the coordination force could be produced between the hydrophilic segments of micelles and Cu^2+^, forming the nuclear and further guiding the growth of MOF nanosheets, and, simultaneously, wrapping the micelles. THF was used to remove the BCP micelles, leaving abundant pores inside the MOF nanosheets (Figure 2f).

**Figure 3:**
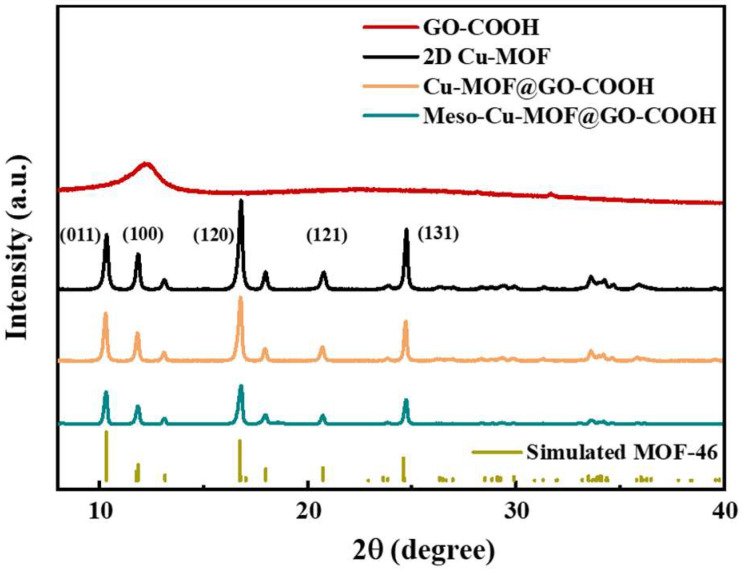
XRD patterns of samples.

To further understand the crystal structure of different samples, XRD analysis was performed. As shown in Figure 3, the peaks at 10.3, 11.8, 16.7, 20.8, and 24.8° correspond to the (001), (100), (120), (121), and (131) crystal planes, respectively, which is basically consistent with those of the simulated MOF-46 crystals, proving the success synthesis of 2D Cu-MOF [42,43]. After combining 2D Cu-MOF with GO-COOH, though the corresponding peak intensity decreased slightly, the peak position of Cu-MOF@GO-COOH showed no obvious difference with that of the 2D Cu-MOF. Similarly, compared with Cu-MOF@GO-COOH, Meso-Cu-MOF@GO-COOH presented a very similar peak position except for a slight decrease in peak intensity, which suggests that the crystal structure of Cu-MOF was not obviously changed during the introduction of the mesoporous structure using a soft template.

**Figure 4:**
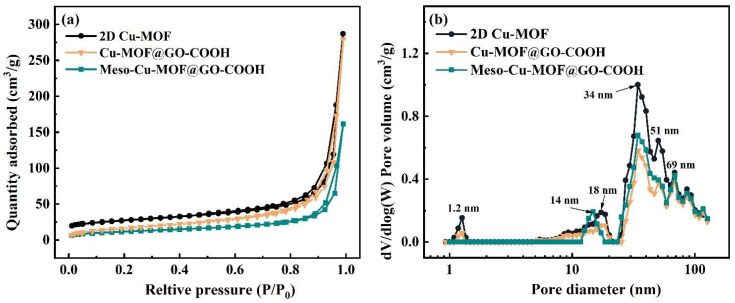
(**a**) N_2_ adsorption–desorption isotherms and (**b**) corresponding BJH pore distribution plots of samples.

The pore characteristics of samples are displayed in Figure 4. The materials exhibit type I isotherms as well as obvious hysteresis loops, indicating the architecture of hierarchical pores. The type I isotherm shape in low pressure suggests the presence of micropores, while the obvious H_3_-type hysteresis loop in higher pressure suggests the presence of large-sized slit pores caused by the accumulation of 2D Cu-MOF nanosheets. As displayed in Figure 4b, similar pore distribution, including micropore (1.2 nm), mesopores (18 and 34 nm), as well as macropores (51 and 69 nm), can be noticed. The proportion of the micropores (1.2 nm) decreased greatly while a new mesopore (14 nm) was formed in Meso-Cu-MOF@GO-COOH. Upon the removal of the BCP micelles, a new mesopore was produced inside the 2D Cu-MOF nanosheets (Figure 2f), which could provide interpenetrated channels for ion transport and subsequently make it easier to access the active sites in MOF.

**Figure 5:**
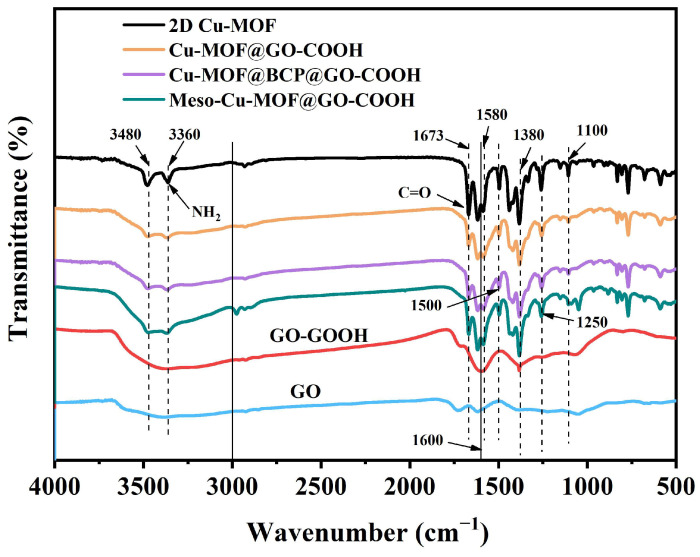
FTIR spectra of samples.

The infrared spectra of samples are shown in Figure 5. Obviously, for GO-COOH, the broad peak at 3666~3245 cm^−1^, corresponding to the stretching vibration of -OH, was strengthened compared with GO, which is associated with the introduction of -COOH [44,45,46,47]. For 2D Cu-MOF and its hybrids, the two peaks at 3364 and 3486 cm^−1^ correspond to the symmetric and asymmetric stretching vibrations of the N-H groups in H_2_BDC-NH_2_, respectively. The peak of C=O in 2D Cu-MOF presented a blue shift to 1669 cm^−1^ compared with that in GO and GO-COOH, which is related to the coordination with Cu^2+^ ions, also proving the success synthesis of 2D Cu-MOF. Because the characteristic functional groups of PS_102_-b-PEO_114_ are similar to those of H_2_BDC-NH_2_, the characteristic peaks of Cu-MOF@BCP@GO-COOH presented no obvious difference to those of Cu-MOF@GO-COOH and Meso-Cu-MOF@GO-COOH. However, in the above-mentioned SEM analysis (Figure 2), the difference in the microstructure of the as-prepared samples has been clearly displayed.

### 3.2. Electrochemical Performance of 2D MOF Hybrid Electrodes

**Figure 6:**
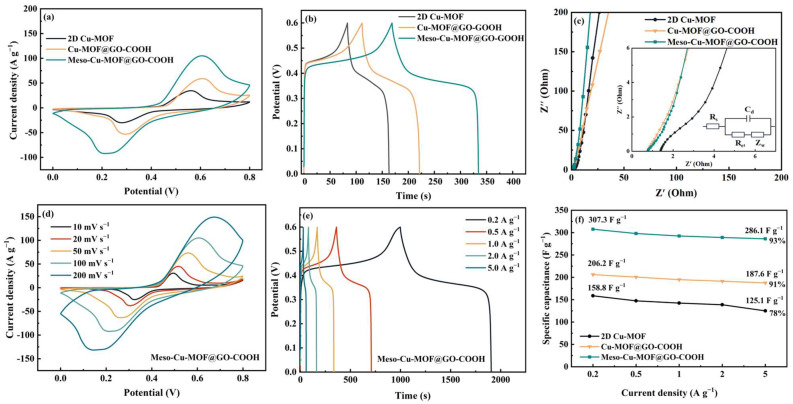
Electrochemical performances of samples: (**a**) CV curves at a scan rate of 100 mV s^−1^, (**b**) GCD curves at a current density of 1 A g^−1^, and (**c**) Nyquist plots of samples. (**d**) CV curves at various scan rates, (**e**) GCD curves at various current densities, and (**f**) the specific capacitance as a function of current density of samples.

The electrochemical performances of the prepared materials are shown in Figure 6. According to the CV and GCD curves, the energy storage process of the hybrids consists of pseudocapacitive and double-layer behaviors. The enclosed region in CV curves (Figure 6a) reveals the ability of the samples to store charges. Furthermore, at a current density of 1 A g^−1^ (Figure 6b), the capacitance values of 2D Cu-MOF, Cu-MOF@GO-COOH, and Meso-Cu-MOF@GO-COOH were calculated to be 141.4, 193.7, and 292.5 F g^−1^ (1 A g^−1^), respectively, which can be explained by the Nyquist plots and equivalent circuit in Figure 6c. Clearly, the two hybrids exhibit a smaller intercept at the real axis compared with 2D Cu-MOF, implying lower internal resistance (*R*s) owed to the introduction of GO-COOH substrate. The pi-pi interaction between 2D Cu-MOF and GO-COOH nanosheets could enhance carrier transport, which is conducive to improving conductivity. Moreover, in the low-frequency region, Meso-Cu-MOF@GO-COOH presents a steeper slope of the straight line than those of the other samples, suggesting faster ion diffusion rates [48,49,50], which is attributed to the convenient channels for ion transport stemming from the interpenetrated holes inside MOF nanosheets. For the equivalent circuit, *R*_s_ means the electrolyte resistance, *C*_d_ presents the double-layer capacitance, *R*_ct_ is the polarization resistance stemmed from charge transfer, and *Z*_w_ is the Warburg resistance associated with ion diffusion. For Meso-Cu-MOF@GO-COOH, with the increased scan rates, the shapes of the CV curves present no obvious change (Figure 6d), suggesting fast electron transport inside the active material [51]. According to the GCD curves (Figure 6e), the specific capacitance values of Meso-Cu-MOF@GO-COOH were calculated to be 307.3 and 286.1 F g^−1^ as the current density increased from 0.2 to 5 A g^−1^, presenting a 93% capacitance retention (Figure 6f). The as-obtained Meso-Cu-MOF@GO-COOH presents enhanced performance compared with some reported Cu-MOF electrodes (Cu_3_(HHTP)_2_, 110–114 F g^−1^ at 0.04–0.05 A g^−1^ [52]; Cu@BTC, 228 F g^−1^ at 1.5 A g^−1^ [53]; and Cu-MOF, 37.91 F g^−1^ at 0.8 A g^−1^ [54], etc.).

To further investigate the cycling stability of the Meso-Cu-MOF@GO-COOH hybrid, 2000 cycles were performed at a current density of 2 A g^−1^ and the results are shown in Figure S3, delivering an 82% capacitance retention.

### 3.3. Energy Storage Mechanism

By integrating GO-COOH into 2D MOF to construct 2D MOF hybrids and subsequently producing pores inside MOF nanosheets, Cu-MOF@GO-COOH and Meso-Cu-MOF@GO-COOH were endowed with enhanced capacitive properties. To further investigate the energy storage mechanism, the *b*-value model [55] and Dunn method [56] were used to reveal the kinetic behaviors. The peak current (*i*) and scan rate (*v*) can be described in the following formula in the *b*-value model:

(5) $$i=a\cdot \nu^{b}$$

Here, *b* is a variable with a range of 0.5~1.0, and the 0.5 value corresponds to the diffusive-controlled behavior, while the 1.0 corresponds to the surface capacitive behavior.

**Figure 7:**
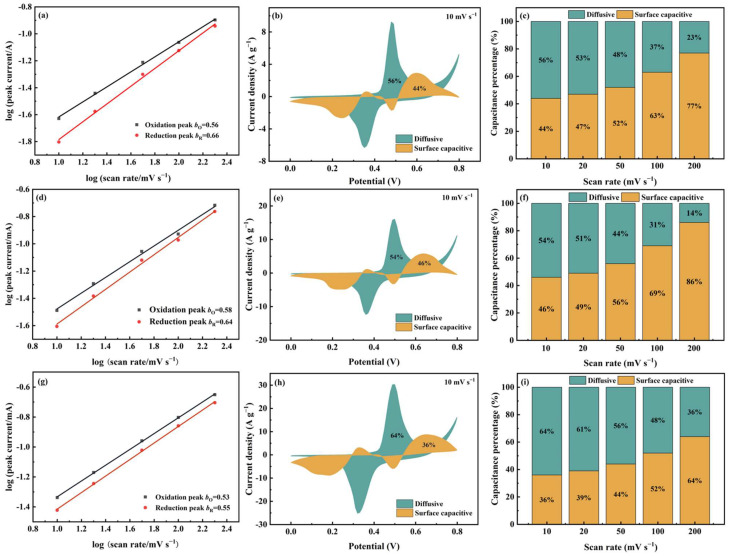
The *b*-value and Dunn model analysis of (**a**–**c**) 2D Cu-MOF, (**d**–**f**) Cu-MOF@GO-COOH, and (**g**–**i**) Meso-Cu-MOF@GO-COOH. (**a**,**d**,**g**) Plots of log(*i*) against log(*v*), (**b**,**e**,**h**) surface capacitive and diffusive contributions at a scan rate of 10 mV s^−1^, and (**c**,**f**,**i**) capacitance contribution ratios at different scan rates.

As shown in Figure 7a,d, for Cu-MOF@GO-COOH, the *b*-values of both oxidation and the reduction peaks are close to those of 2D Cu-MOF, implying that the two samples present similar charge-storage behaviors. Differently, the *b*-values of Meso-Cu-MOF@GO-COOH are closer to the critical value of 0.5, indicating that the diffusive-controlled behavior is enhanced, which can be confirmed by the Dunn method’s results. The contributions of the two charge-storage behaviors can be identified via the Dunn model [57,58]:

(6) $$i(V)=k_{1}\nu +k_{2}\nu^{0.5}$$

where *k*_1_*v* and *k*_1_*v*^0.5^ correspond to the surface capacitive current and diffusive current, respectively, by which the charge-storage process of electrode materials can be investigated. Clearly, at a scan rate of 10 mV s^−1^ (Figure 7b,e,h), the surface capacitive ratios of 2D Cu-MOF, Cu-MOF@GO-COOH, and Meso-Cu-MOF@GO-COOH are 44%, 46%, and 36%, respectively. For 2D Cu-MOF and Cu-MOF@GO-COOH, the surface capacitive percentages increased greatly with the increased scan rates, obtaining 77% and 86%, respectively, at a scan rate of 200 mV s^−1^. Utilizing GO-COOH to construct 2D MOF hybrids accelerates the charge transfer, thus lowering the internal resistance (Figure 6c), and thereby enhancing the surface capacitive behavior, which may be the main reason for the improved capacitance value of Cu-MOF@GO-COOH. Notably, for Meso-Cu-MOF@GO-COOH, even when the scan rate reached 50 mV s^−1^, the ratio of diffusive current was still higher than that of surface capacitive current, implying the dominant diffusion-controlled behavior. Owing to the interpenetrated pores, ions could diffuse adequately inside Meso-Cu-MOF@GO-COOH, allowing the active sites to be utilized more fully and conducting sufficient redox reactions inside MOF nanosheets. Thus, benefiting from the enhanced diffusion behavior, the electrochemical performance of Meso-Cu-MOF@GO-COOH was further improved.

### 3.4. Electrochemical Performance of ASC Device

**Figure 8:**
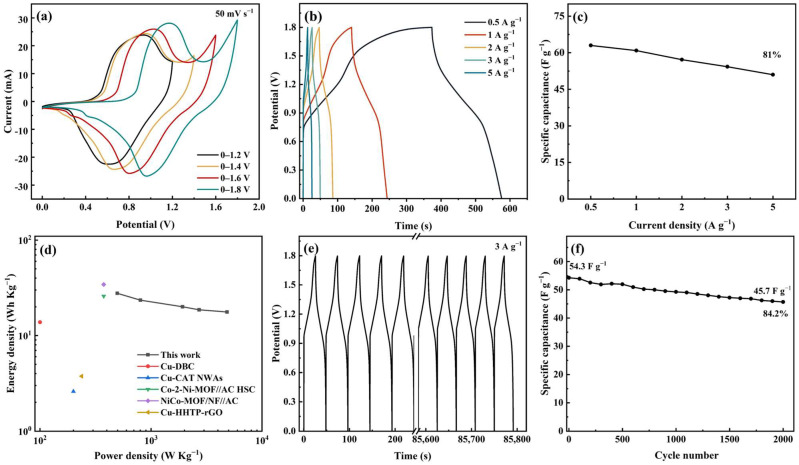
Electrochemical performances of the ASC device. (**a**) CV curves at different potential windows, (**b**) GCD curves at different current densities, (**c**) specific capacitance under various current densities, (**d**) Ragone plots, (**e**) cycling GCD curves, and (**f**) cycling performance.

An ASC device was prepared using Meso-Cu-MOF@GO-COOH and activated carbon (AC) as the positive and negative electrodes, respectively. Combining the capacitance values of AC, calculated via the GCD curves, with the potential ranges of two electrodes (Figure S4), the optimal mass ratio (*m*_+_/*m*_−_) of 1.2 was chosen to prepare the ASC device. Furthermore, CV tests under various voltage windows showed that the curve shape presented no deformation even at 1.8 V (Figure 8a), exhibiting a broad working voltage window. With the scan rate was increased to 100 mV s^−1^ (Figure S4), no obvious changes can be noticed, indicating fast charge transfer and ion transport.

The capacitance values corresponding to 0.5, 1, 2, 3, and 5 A g^−1^ were calculated to be 63, 60.9, 57.2, 54.3, and 50.9 F g^−1^, respectively, and the 81% capacitance retention rate shows a relatively good performance rate (Figure 8b,c). The device delivers a maximum energy density of 27.7 Wh kg^−1^ at a power density of 496.8 W kg^−1^, which are figures comparable to or surpassing many of the recently reported MOF devices mentioned in Figure 8d [51,59,60,61,62]. Moreover, the cycle performance was investigated at a current density of 3 A g^−1^, delivering a capacitance retention of 84.2% after 2000 cycles (Figure 8e,f).

## 4. Conclusions

Here, a hierarchical Meso-Cu-MOFs@GO-COOH was synthesized and used as supercapacitor electrode. The introduction of GO-COOH accelerates electron transfer, lowers the internal resistance of the hybrid, and thus enhances the charge storage capacity by promoting the surface capacitive behavior. Additionally, the interpenetrated mesopores inside MOF sheets optimize the diffusion paths of ions, improving the energy storage capacitance via enhancing the diffusive behavior. The Meso-Cu-MOFs@GO-COOH exhibited an increased capacitance of 292.5 F g^−1^ (1 A g^−1^), which is 1.5 and 2.1 times as high as that of Cu-MOFs@GO-COOH and 2D Cu-MOF, respectively. When the current density increased from 0.2 to 5 A g^−1^, an excellent capacitance retention of 93% was obtained. Furthermore, the assembled Cu-MOF@GO-COOH//AC supercapacitor exhibited a capacitance of 63 F g^−1^ (0.5 A g^−1^), an energy density of 27.7 Wh kg^−1^, and a power density of 496.8 W kg^−1^, showing its practical potential in energy storage applications.

## Data Availability

The original contributions presented in this study are included in the article and Supplementary Material. Further inquiries can be directed to the corresponding authors.

## Supplementary Materials

The following supporting information can be downloaded at: https://www.mdpi.com/article/10.3390/nano15211628/s1, Figure S1: SEM of BCP@GO-COOH samples. (a) PS_70_-BCP@GO-COOH, (b) PS_90_-BCP@GO-COOH, (c) PS_102_-BCP@GO-COOH and (d) PS_150_-BCP@GO-COOH; Figure S2: TEM image of Cu-MOF@GO-COOH; Figure S3: Cycling test of Meso-Cu-MOF@GO-COOH: (a) GCD curves at a current density of 2 A g-1 and (b) cycling capacitance; Figure S4: (a) GCD curves of AC electrode, (b) CV curves of AC and Meso-Cu-MOF@GO-COOH, and (c) CV curves of Meso-Cu-MOF@GO-COOH at different scan rates.
