# Supplementary material for: Hierarchical 2D Cu-MOF@Graphene-Based Hybrids for Supercapacitor Electrodes
Source: Nanomaterials (Basel). 2025 Oct 25;15(21):1628. doi: 10.3390/nano15211628 (PMC12608233; doi:10.3390/nano15211628)
Supplement: Supplementary file 1 [file nanomaterials-15-01628-s001.zip › nanomaterials-3938258-supplementary.pdf]

Supporting Information

Hierarchical 2D Cu-MOF@Graphene-based hybrids for supercapacitor electrodes

Mengkun Yang [a], Yongqiang Zhang [a], Wenjie Li [a], Pingwei Ye [b, *], Yijing Nie [a], Maiyong Zhu [a], Sumin Li [a, *]

[a] *School of Materials Science & Engineering, Jiangsu University, Zhenjiang 212013, China*

[b] *State Key Laboratory of NBC Protection for Civilian, Beijing 102205, China*

[*] *Corresponding authors. E-mail addresses:* li_sm@ujs.edu.cn (S. Li), yepw2001@163.com (P. Ye)

Materials synthesis

Synthesis of 2D Cu-MOF. 20 mg $Cu(OAc)_2•H_2O$ was dissolved in a mixed solvent containing 2 mL of N, N-dimethylformamide (DMF) and 4 mL of ethanol absolute (EtOH) to prepare a metal salt solution. Meanwhile, 20 mg of $H_2BDC-NH_2$ was dissolved in another mixed solution of DMF (10 mL) and EtOH (5 mL) to obtain an organic ligand solution. The salt solution was then slowly added to the organic solution under stirring and stirred continuously for 30 min. After that, the product was collected and washed with DMF and EtOH, and then dried at 60 °C for 24 h.

Synthesis of GO-COOH. Under stirring, 168 mg of NaOH and 140 mg of chloroacetic acid were added to 300 mL of aqueous solution of GO (0.5 mg mL^{-1}). Then, the mixture was kept at 70 °C for 1 h. Then, the mixture was neutralized (pH=7) with 1 M hydrochloric acid (HCl). The product was collected, washed with deionized water, and then freeze-dried to obtain GO-COOH sample.

Synthesis of BCP@GO-COOH. 10 mg of PS_{102}-b-PEO_{114} copolymer was added to 10 mL of DMF and sonicated for 10 min. Then, 1 mL of EtOH was slowly added under stirring. After that, 4 mL of EtOH was also added with a stirring for 30 min to obtain a block copolymer (BCP) solution. Subsequently, 10 mg of GO-COOH was added to the BCP solution and sonicated for 60 min. At last, the solution was stirred for 30 min to obtain the BCP@GO-COOH solution.

Preparation of working electrodes

Working electrodes were prepared as follows. Acetylene black (10 wt.%), polyvinylidene fluoride (PVDF, 10 wt.%) and active materials (80 wt.%) were mixed with N-methyl 2-pyrrolidone to form a

homogeneous paste. Afterwards, the paste was coated on a piece of Ni foam (1×1 cm^2) current collector, and then dried at 80 °C for 12 h to obtain a working electrode.

Results and analysis

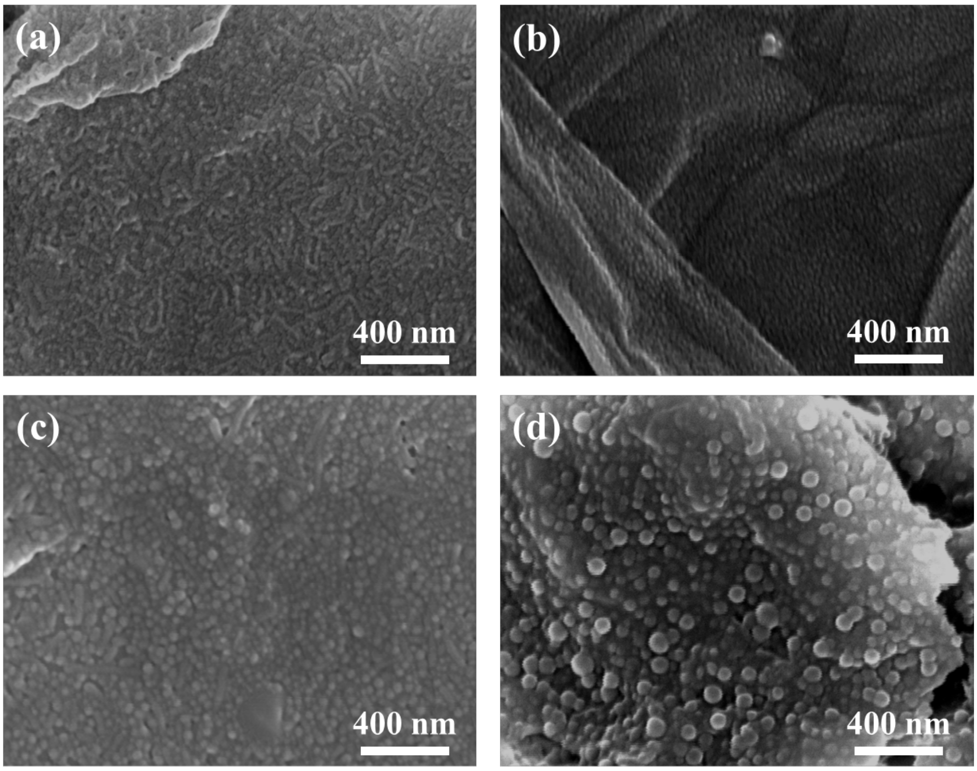

Figure S1. SEM of BCP@GO-COOH samples. (a) PS_{70}-BCP@GO-COOH, (b) PS_{90}-BCP@GO-COOH, (c) PS_{102}-BCP@GO-COOH and (d) PS_{150}-BCP@GO-COOH.

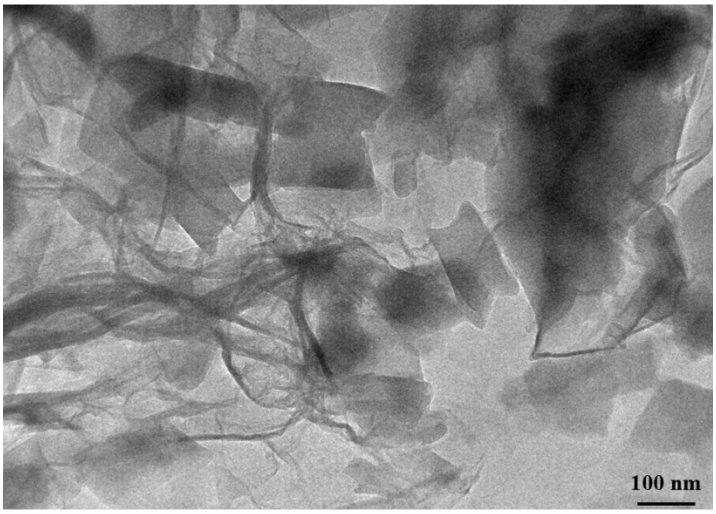

Figure S2. TEM image of Cu-MOF@GO-COOH.

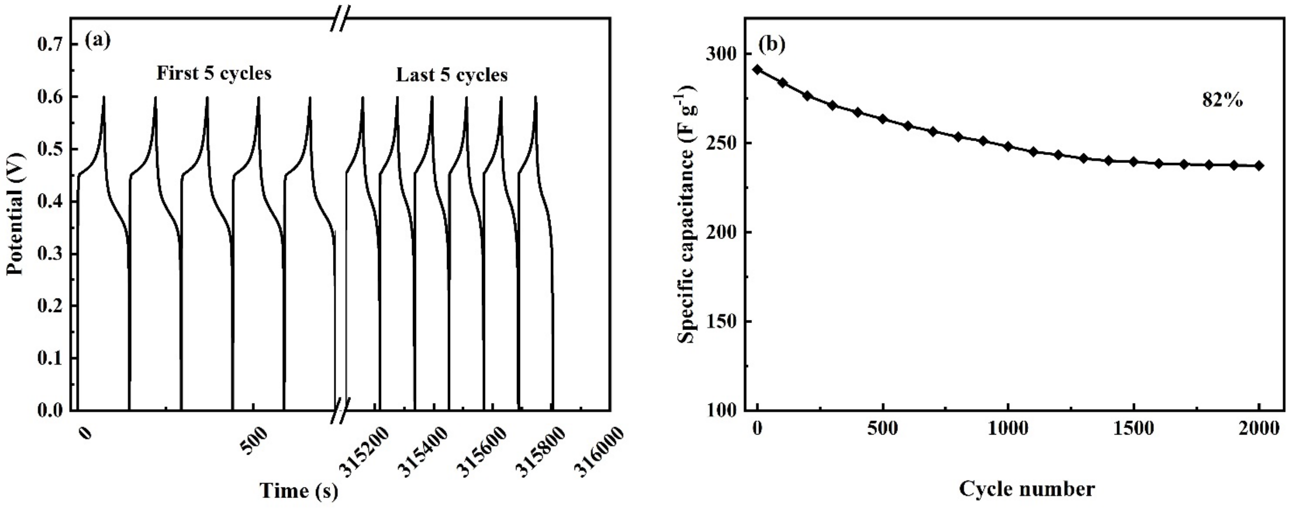

Figure S3. Cycling test of Meso-Cu-MOF@GO-COOH: (a) GCD curves at a current density of 2 A g^{-1} and (b) cycling capacitance.

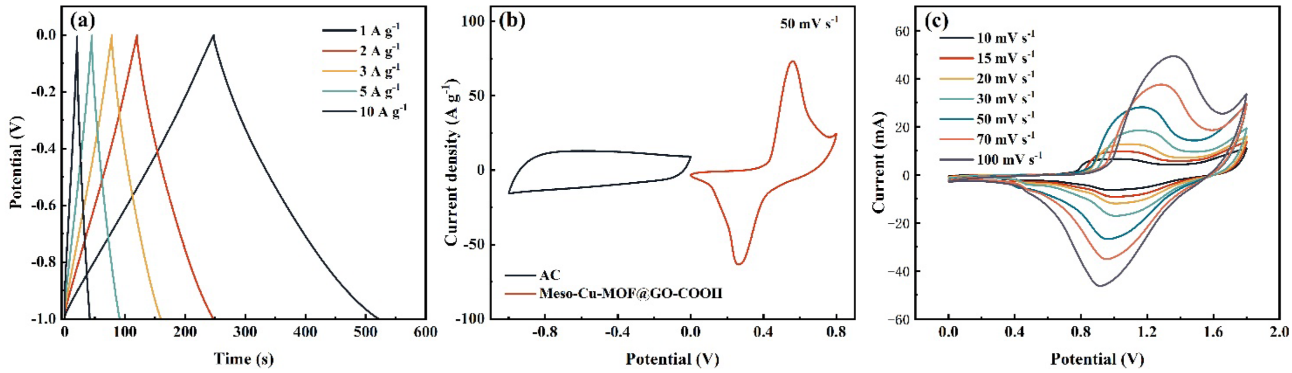

Figure S4. (a) GCD curves of AC electrode, (b) CV curves of AC and Meso-Cu-MOF@GO-COOH, and (c) CV curves of Meso-Cu-MOF@GO-COOH at different scan rates.
